# Exploring the Therapeutic Effect of *Polygonatum cyrtonema* Polysaccharides in Reversing D-Galactose (D-Gal)-Mediated Cardiac Aging

**DOI:** 10.3390/nu18091390

**Published:** 2026-04-28

**Authors:** Yaxian Wang, Limin Ouyang, Ximin Wu, Quan Tao, Yue Zhou, Binrui Yang, Lu Chen, Lu Zhang, Huali Wu, Doudou Huang, Liang Chen, Yiming Li

**Affiliations:** 1 School of Pharmacy, Shanghai University of Traditional Chinese Medicine, Shanghai 201203, China; somo_amour@163.com (Y.W.); wxmtcm@126.com (X.W.); tq2021431047@163.com (Q.T.); zhoudiandiana@163.com (Y.Z.); crystal0429@126.com (L.C.); wu5254307@163.com (H.W.); hdd890920@163.com (D.H.); 2 Nutrilite Antiaging Research Center, Amway (Shanghai) Innovation & Science Co., Ltd., Shanghai 201203, China; limin.ouyang@amway.com (L.O.); binrui.yang@amway.com (B.Y.); lynn.zhang@amway.com (L.Z.)

**Keywords:** *Polygonatum cyrtonema*, polysaccharides, cardiac aging, cellular senescence, oxidative stress

## Abstract

Background/Objectives: Cardiac aging is characterized by increased oxidative stress and mitochondrial dysfunction in cardiomyocytes, leading to structural remodeling and functional decline. *Polygonatum cyrtonema* polysaccharides (PCPs), the principal active components derived from *Polygonatum cyrtonema*, exhibit well-documented antioxidant and anti-inflammatory effects. Despite this, their protective role against cardiac aging and the underlying molecular mechanisms remain largely unexplored. This study aimed to investigate the protective action of PCPs against D-galactose(D-gal)-induced cardiomyocyte senescence. Methods: In vitro, a cellular senescence model was established in H9c2 cardiomyocytes by D-gal induction to elucidate the effects of PCPs on senescence and mitochondrial dysfunction. In vivo, a mouse aging model was generated in C57BL/6J mice via continuous intraperitoneal injection of D-gal for three months to evaluate the ameliorative effects of PCPs on aging phenotypes and cardiac function. Results: PCPs enhanced the antioxidant capacity of cardiomyocytes, improved energy metabolism homeostasis, maintained mitochondrial integrity, thereby synergistically regulating key aging-related signaling pathways such as suppressing overactivation of the p53/p21 axis and downregulating the expression of the senescence-associated secretory phenotype, thereby effectively mitigating myocardial injury and delaying cellular senescence. Conclusions: This study demonstrates the anti-cardiac aging effects of PCPs at both cellular and animal levels, confirming that they protect cardiomyocytes by antagonizing oxidative stress, suppressing the p53/p21 pathway, and improving mitochondrial function. These findings provide an experimental basis for developing PCPs as a naturally sourced intervention against cardiac aging.

## 1. Introduction

Aging is an inevitable physiological process in life, accompanied by the gradual decline of cellular, tissue, and organ functions. This leads to increased susceptibility to chronic diseases and a corresponding rise in mortality risk. With advances in socio-economic conditions and scientific technology, the global average life expectancy has risen from approximately 52 years in 1960 to about 73 years today. According to United Nations data, the global population aged 60 and above reached 1.2 billion in 2025, accounting for approximately 14.7% of the total population. By 2050, this number is projected to further increase to 1.6 billion, representing approximately 20% of the global population [1]. Population aging continues to intensify worldwide, with the proportion of older adults steadily rising in both developed and many developing countries [2]. Although human life expectancy has increased significantly, the proportion of healthy life expectancy within the overall lifespan remains relatively low [3], indicating that many people still face health challenges in their later years. Therefore, how to achieve healthy aging while extending lifespan has become an important topic in scientific research.

The heart is a key organ for maintaining normal human physiological functions, and its aging is a complex, irreversible biological process [4] that profoundly affects lifespan, health status, and quality of life. With the global aging population intensifying, the incidence and mortality of cardiovascular diseases (CVDs) continue to rise, imposing prolonged suffering on the elderly and a substantial socio-economic burden. Aging is an independent risk factor for CVDs [5], highlighting the close link between cardiac aging and cardiovascular pathogenesis. With advancing age, the heart undergoes progressive structural and functional degenerative changes, mainly cardiac hypertrophy, impaired systolic and diastolic function, and reduced pumping capacity. These changes significantly increase the risk of age-related cardiac conditions such as heart failure, arrhythmias, and coronary artery disease [6]. Cardiac aging is driven by a complex interplay of molecular and cellular alterations, including maladaptive cellular metabolism, impaired autophagy, cardiomyocyte dysfunction, reduced angiogenesis, and exacerbated tissue fibrosis [7]. A hallmark of these changes is the progressive loss of proliferative capacity and dysregulated cell cycle control in both cardiomyocytes and non-cardiomyocytes, culminating in irreversible cell cycle arrest [8]. Furthermore, the accumulation of DNA damage and increased reactive oxygen species (ROS) due to oxidative stress are key mechanisms triggering cellular senescence [9], which is closely linked to cardiac aging. Notably, senescence of different cardiac cell types causes distinct CVDs: endothelial cell senescence promotes atherosclerosis, cardiomyocyte senescence increases the risk of myocardial infarction, and fibroblast senescence accelerates cardiac fibrosis [10]. Therefore, elucidating the molecular and cellular mechanisms of cardiac aging and developing effective interventional strategies have become major focuses in modern medical research—critical for reducing the burden of cardiovascular morbidity and mortality in the elderly amid global aging.

*Polygonati rhizoma* is the dried rhizome of *Polygonatum sibiricum Red.* (family *Liliaceae*). As defined by the 2020 edition of the Pharmacopoeia of the People’s Republic of China, this medicinal material encompasses the dried rhizomes of *Polygonatum sibiricum Red.*, *Polygonatum kingianum Coll. et Hemsl.*, and *Polygonatum cyrtonema Hua*. Known for its sweet taste, it has been historically valued in traditional practices for its potential to promote health and longevity, and is recognized as a renowned traditional medicinal substance with both edible and medicinal uses in China [11]. It demonstrates a broad spectrum of pharmacological applications and biological activities, including antioxidant [12], anti-aging [13], anti-osteoporosis [14], immunoenhancing [15], anti-diabetic [16], anti-fatigue [17], and anti-tumor [18] effects. These diverse bioactivities are attributed to the presence of various constituents, such as alkaloids, flavonoids, steroidal saponins, lignans, amino acids, and polysaccharides.

PCPs one of the primary active constituents of *Polygonati rhizoma*, exhibit a wide range of biological activities, including antitumor, antioxidant, anti-aging, anti-inflammatory, and immunomodulatory effects [19]. Its structure is complex, primarily composed of monosaccharides such as glucose, fructose, galactose, and arabinose [20]. As a natural macromolecule, PCPs have garnered significant attention for their potential applications in functional foods and medicine, with their anti-aging effects being particularly notable. Current research indicates that PCPs play a positive role in delaying tissue senescence. For instance, in a cellular model, PCPs alleviated D-gal-induced oxidative stress and mitochondrial dysfunction in mouse myotube C2C12 cells [21]. They improved mitochondrial function and delayed skeletal muscle aging by maintaining mitochondrial morphology, reducing mitochondrial-associated membrane formation, and regulating calcium homeostasis [21]. In animal studies, Zheng et al. [22] found that PCPs mitigated oxidative stress, enhanced learning and memory capabilities, and reversed pathological changes in renal tissue in D-gal-induced aging rats by modulating the Klotho-FGF23 endocrine axis. Furthermore, Bian et al. [23] demonstrated that PCPs ameliorate oxidative stress and memory impairment in aging mice by preserving synaptic plasticity and reducing neuronal damage.

Studies using the *Caenorhabditis elegans* aging model have further revealed that PCPs exert anti-aging effects by modulating the insulin signaling pathway. Specifically, PCPs significantly downregulated the expression of the pro-aging factor DAF-2, while upregulating the expression of the transcription factor DAF-16 and superoxide dismutase SOD-3, reducing the accumulation of lipofuscin in worms, thereby delaying aging and extending lifespan [24].

Beyond these mechanisms, PCPs also delay age-related functional decline by regulating immune and inflammatory responses. Research has shown that PCP treatment increases the thymic and splenic indices in mice, promotes splenocyte proliferation, and enhances macrophage phagocytic function, thereby improving the overall immune status of the organism [25]. Regarding inflammation regulation, PCPs inhibit the expression of inflammatory cytokines TNF-α, IL-1β, IL-6, and IL-8 via the TLR4/MyD88/NF-κB signaling pathway, consequently alleviating lipopolysaccharide (LPS)-induced acute lung injury [26]. Furthermore, in high-glucose-stimulated human retinal pigment epithelial cells (ARPE-19), PCPs effectively suppressed the release of pro-inflammatory factors and alleviated oxidative stress and apoptosis [27]. These findings collectively demonstrate, from multiple perspectives, the significant potential of PCPs in intervening in aging and its related pathological processes.

While the anti-aging effects of PCPs have been extensively demonstrated in vivo and across multiple tissues—including skeletal muscle, neural tissue, kidney, and the immune system—their potential role in protecting against cardiac aging remains largely unexplored. No studies to date have systematically investigated whether PCPs can attenuate cardiac aging or elucidated the underlying mechanisms. Therefore, the present study aims to address this gap through a comprehensive investigation combining in vitro and in vivo experiments, providing both mechanistic insights and a theoretical foundation for the potential application of PCPs in preventing age-related cardiac dysfunction. In the in vitro phase, we employed a D-gal-induced aging model in rat cardiomyocytes to systematically evaluate the direct effects of PCPs on key cardiac aging-related phenotypes, such as cellular senescence and oxidative stress, and to preliminarily elucidate the underlying molecular pathways. For the in vivo phase, an aging model was established in C57BL/6J mice via continuous three-month intraperitoneal injection of D-gal, followed by oral administration of different doses of PCPs. The anti-cardiac aging effects were comprehensively assessed at multiple levels, including cardiac function, tissue morphology, and molecular markers. This study is intended to provide new theoretical insights and potential intervention strategies for the prevention and treatment of age-related cardiac diseases, while also laying an experimental foundation for the future clinical translation of PCPs.

## 2. Materials and Methods

### 2.1. Extraction of Polygonatum cyrtonema Polysaccharides

A total of 10 kg crude *Polygonatum cyrtonema* was extracted using 90% ethanol (1:10) under reflux for 2 h. The above ethanol extract was subjected to D101 resin and eluted with 40 L distilled water (L01). Then the *Polygonatum cyrtonema* dreg was refluxed using 12-fold distilled water twice (2 h each), and the extract was collected (L02). After that, L01 and L02 were combined and 95% ethanol was added to the extract to achieve an ethanol concentration of 80%. The resulting precipitate was collected by filtration to obtain crude PCPs. The aforementioned polysaccharides were subsequently dissolved in a suitable volume of water, and proteins were removed using the Sevage method. The aqueous solution was then subjected to organic solvent recovery, followed by lyophilization to yield the total PCPs.

### 2.2. Measurement of the Content, Molecular Weight and Monosaccharide Composition

A high-performance gel permeation chromatography (HPGPC) approach was used for assessing PCP molecular weight on an Agilent 1100 HPLC machine (Agilent Technologies, Santa Clara, CA, USA). Pullulans of known molecular weights (6.1 kD up to 642 kD) were used for standard curve calibration, and 0.2 mol/L NaCl was used for column elution at a 0.8 mL/min flow rate. Samples were prepared at 2.0 mg/mL, with 20 μL aliquot being injected for analysis.

PCPs were hydrolyzed with 2 M trifluoroacetic acid (TFA) at 120 °C for 2 h. Methanol evaporations were then performed repeatedly until TFA was fully removed, and remaining residues were resuspended in water, reduced at room temperature for 3 h using NaBH4 neutralized using acetic acid (AcOH), and evaporated until dry. Next, acetic anhydride (Ac2O) was used to treat samples at 100 °C for 1 h in order to achieve acetylation, with HP-5MS (60 m × 0.25 mm × 0.25 μm) (Agilent Technologies, Santa Clara, CA, USA) as GC–MS analyses. For such monosaccharide GC–MS analyses, the program settings were as follows: rising from 120 °C to 190 °C at 2 °C/min, rising to 250 °C at 2 °C/min, followed by a 5 min hold.

The *Polygonatum cyrtonema* Hua. was sourced from the Nutrilite Antiaging Research Center, Amway (Shanghai) Innovation & Science Co., Ltd. (Shanghai, China) The material was harvested in October 2022 in Jinzhai, Anhui Province, with the batch number TS22C189. Authentication was performed by Dr. Xuefei Cai of the Nutrilite Antiaging Research Center, Amway (Shanghai) Innovation & Science Co., Ltd.

### 2.3. Animals

Eight-week-old healthy male C57BL/6J mice were purchased from Vital River Laboratory Animal Technology Co., Ltd. (Beijing, China). All mice were housed under standardized environmental conditions, where the temperature was maintained at 22 °C (±2 °C), a 12 h light/dark cycle was adopted, and the relative humidity was controlled at 40–70%. The experimental unit was the individual mouse. Each mouse was housed separately in a cage (or in groups of 5 per cage) and received independent treatment (D-gal injection and PCP administration). To enhance animal welfare and minimize aggressive interactions among cage mates, each cage was enriched with a shelter and gnawing sticks. All animals had adlibitum access to food and water throughout the experiment. The inclusion and exclusion criteria were established a priori as follows: all mice were required to be healthy male C57BL/6J mice aged 8 weeks after one week of acclimatization. Animals were to be excluded if they exhibited severe weight loss (>20% of initial body weight), visible signs of illness or distress, or if they died before the experimental endpoint. The successful establishment of the cardiac aging model was confirmed by significant deterioration in cardiac function observed via echocardiography, at which point the experimental endpoint was reached.

All animal experiments were approved by the Institutional Animal Care and Use Committee (IACUC) of Shanghai University of Traditional Chinese Medicine. The project application code is No. PZSHUTCM2410180002, approved on 26 September 2025.

### 2.4. Model Building and Drug Delivery Methods

All experimental procedures adhered to the 3R principles (Replacement, Reduction, Refinement) for ethical animal research. The sample size was determined based on several experimental studies [28,29,30] and preliminary data from our laboratory. Accordingly, a total of 20 mice were divided into 4 groups (*n* = 5 per group), which was deemed sufficient to ensure adequate statistical power. Following a one-week acclimatization period, the twenty mice were randomly assigned into four groups using a random number table (*n* = 5 per group): (1) control group; (2) D-gal model group; (3) PCP low-dose group; and (4) PCPs high-dose group. The dosage and administration schedule of D-gal and PCP were determined according to previously published studies [21,31]. With the exception of the control group, which received daily intraperitoneal injections of an equal volume of normal saline, all other groups were injected daily with 400 mg/kg D-gal to induce aging. Concurrently, the low- and high-dose PCP groups received daily oral gavage of PCPs at 200 mg/kg and 400 mg/kg, respectively, while the control and D-gal model groups were administered an equal volume of vehicle (normal saline) via gavage. Body weight was recorded on a weekly basis, and food intake was measured every two weeks. To minimize potential confounding effects, drug administration was performed in a fixed daily order, while the order of cage handling was rotated daily. Cage positions on the racks were rotated every two weeks throughout the experiment. For outcome assessments, the order of measurements was randomized across groups, and all analyses were conducted by researchers blinded to group allocation whenever feasible. Meanwhile, the fur condition and behavioral changes in the animals were monitored continuously, and echocardiographic examinations were conducted at week 12. For transthoracic echocardiography, mice were anesthetized using a gas anesthesia machine (vaporizer system) with 2% isoflurane delivered in oxygen via a nose cone. The depth of anesthesia was monitored by the absence of pedal reflexes. Echocardiographic images were acquired using a Vevo 2100 high-resolution imaging system (VisualSonics, Toronto, ON, Canada). Data analysis was performed using the VevoLab 3.0.0 software (VisualSonics). The experiment was conducted over a 12-week period. All mice remained healthy throughout the study, and accordingly, no data exclusion was necessary for any of the analyses. At the endpoint, all mice were euthanized by cervical dislocation under deep anesthesia (induced by 5% isoflurane and maintained with 2% isoflurane) and cardiac perfusion was performed immediately. Cardiac tissues were subsequently collected and processed as follows: one portion was fixed in paraformaldehyde for histopathological analysis. Given the limited size of cardiac tissue samples, specimens from three randomly selected mice per group were snap-frozen in liquid nitrogen and stored at −80 °C for subsequent biological assays. Throughout the experimental period, no expected or unexpected adverse events, including mortality, abnormal behavior, or signs of severe distress, were observed in any of the animals.

### 2.5. Cell Culture and Treatment

H9c2 cardiomyocytes, obtained from the National Collection of Authenticated Cell Cultures at the Chinese Academy of Sciences, were maintained in DMEM (MA0212, Meilunbio, Dalian, China) containing 10% fetal bovine serum (PWL001, Meilunbio, Dalian, China) and 1% penicillin/streptomycin (C100C5, New Cell & Molecular Biotech, Suzhou, China). The cells were divided into four experimental groups: control, D-gal, PCPs(L), and PCPs(H). Twenty-four hours after seeding, the culture medium in all groups except the control was replaced with medium containing 150 mM D-galactose (G5388, Sigma, St. Louis, MO, USA). The PCPs(L) and PCPs(H) groups were then treated with 100 μg/mL and 200 μg/mL of PCPs, respectively, for 48 h.

### 2.6. Cell Viability Assay

H9c2 cardiomyocytes were seeded in a 96-well culture plate at a density of 5 × 10^4^ cells per well and treated with various concentrations of PCPs for 48 h. Cell viability was assessed using a Cell Counting Kit-8 (CCK-8, MA0218, Meilunbio, Dalian, China). Briefly, 100 μL of CCK-8 reagent (10 μg/mL) was added to each well, followed by incubation at 37 °C for 1 h. The optical density (OD) at 450 nm was then measured using a microplate reader (BioTek, Winooski, VT, USA).

### 2.7. Cell Senescence Assay

Cell senescence was detected using a senescence-associated β-galactosidase (SA-β-Gal) staining kit (C0602, Beyotime, Shanghai, China) according to the manufacturer’s instructions. Briefly, H9c2 cardiomyocytes were seeded into 12-well plates at a density of 5 × 10^4^ cells per well and cultured overnight in a 5% CO_2_ incubator at 37 °C. The cells were then divided into four groups: a control group, a model group treated with 150 mM D-gal to induce senescence, and two intervention groups treated with 150 mM D-gal plus either 100 μg/mL or 200 μg/mL PCPs. After 48 h of treatment, the culture medium was aspirated and the cells were gently washed once with phosphate-buffered saline (PBS). The cells were fixed with 500 μL of β-galactosidase staining fixative for 15 min at room temperature. After fixation, the cells were washed three times with PBS (5 min per wash). Subsequently, 500 μL of staining working solution was added to each well, and the plates were sealed with plastic wrap to prevent evaporation and incubated overnight at 37 °C (without CO_2_). Stained cells were imaged using an inverted microscope, and the percentage of SA-β-Gal-positive cells was quantified using ImageJ 1.x software (National Institutes of Health, Bethesda, MD, USA).

### 2.8. Determination of Mitochondrial Membrane Potential (MMP) Detection

The MMP of H9c2 cardiomyocytes was assessed using tetramethylrhodamine, ethyl ester (TMRE, C2001S, Beyotime, Shanghai, China), a fluorescent dye that accumulates in mitochondria in a membrane potential-dependent manner. Briefly, cells cultured in 6-well plates were incubated with 1 mL of TMRE working solution at 37 °C for 30 min. After incubation, the staining solution was aspirated, and the cells were washed twice with pre-warmed culture medium. Subsequently, 2 mL of fresh pre-warmed medium was added, and fluorescence was immediately visualized under a fluorescence microscope. The MMP was quantified based on fluorescence intensity and expressed as a percentage relative to the untreated control group.

### 2.9. ROS Detection

Intracellular ROS levels were measured using the fluorescent probe DCFH-DA. The cell-permeable, non-fluorescent DCFH-DA is hydrolyzed by intracellular esterases to DCFH, which is trapped within the cells. In the presence of ROS, DCFH is oxidized to the highly fluorescent compound DCF, allowing for the quantification of intracellular ROS levels based on fluorescence intensity. Briefly, DCFH-DA (S0033S, Beyotime, Shanghai, China) was diluted 1:1000 in culture medium to a final concentration of 10 μmol/L. After removing the culture medium, the cells were incubated with the diluted DCFH-DA solution (≥1 mL per well of a 6-well plate) at 37 °C for 20 min. Subsequently, the cells were washed three times with PBS to remove any extracellular probe. Fluorescence was immediately visualized and captured using a laser scanning confocal microscope.

### 2.10. Western Blot Assay

H9c2 cardiomyocytes and cardiac tissues were lysed with RIPA buffer (P1003B, Beyotime, Shanghai, China) containing protease inhibitor and phosphatase inhibitor (P001, P003, New Cell & Molecular Biotech, Suzhou, China) according to the manufacturer’s instructions. Nuclear protein was extracted using a nuclear protein extraction kit (R0050, Solarbio, Beijing, China). After centrifugation (12,000× *g*, 10 min, 4 °C), the supernatant was transferred to a new centrifuge tube, and the protein concentrations were evaluated using a bicinchoninic acid (BCA) protein concentration assay kit (P0011, Beyotime, Shanghai, China). The proteins were added to 5× loading buffer (1LT101S, Epizyme, Shanghai, China) and denatured by heating at 100 °C for 10 min. A total of 30 μg of protein samples was separated on a 10% sodium dodecylsulfate polyacrylamide gel and transferred to polyvinylidene difluoride membrane (Millipore Corporation, Billerica, MA, USA). The membranes were blocked in TBST containing 5% skim milk at room temperature for 1 h and were subsequently incubated with primary antibodies at 4 °C overnight. On the second day, the primary antibodies were collected, washed three times with 5% TBST, and incubated with goat anti-mouse or anti-rabbit IgG HRP for 1 h at room temperature. An ECL Kit (MA0186, Meilunbio, Dalian, China) was used to visualize the protein bands, and the chemiluminescent signals on of the membranes were detected by a Tanon 4600 (Tanon, Shanghai, China). The optical density of the bands were analyzed with Image J software. The primary antibodies were shown as Table S1. β-Tubulin and Histone H3 were used as internal controls. Quantification of the total protein was determined relative to β-Tubulin and Histone H3, and the phospho-protein level was determined relative to the total protein. The protein amounts in the experimental groups were calculated for each experiment and normalized to control values to avoid within-group variations.

### 2.11. Real-Time Fluorescence Quantitative PCR

Total RNA was isolated from heart tissue using a RNA Purification Kit (B0004D, EZBiosience, Suzhou, China) according to the manufacturer’s protocol. Complementary DNA (cDNA) was synthesized from 2000 ng of total RNA with the microRNA Reverse Transcription Kit PLUS (EZB-miRT2-plus, EZBiosience, Suzhou, China). Quantitative real-time polymerase chain reaction (RT–qPCR) was performed using a reaction mixture containing Color SYBR Green qPCR Mix (A0012, EZBiosience, Suzhou, China). The results were calculated using the 2^−ΔΔct^ relative quantification method and normalized to the GAPDH gene. The sequences of the primer pairs are shown in Table S2.

### 2.12. Wheat Germ Agglutinin Staining

Freshly collected heart tissue was fixed in 4% paraformaldehyde at 4 °C for 24–48 h to preserve its structure. The fixed tissue was dehydrated in a series of ethanol solutions. Then, it was cleared with xylene and embedded in paraffin. Addtionally, 4–6 µm-thick sections were cut using a microtome and placed on glass slides. Paraffin was removed with xylene, and the tissue was rehydrated through graded ethanol solutions. Non- specific binding sites were blocked with 5% BSA. The WGA conjugate was diluted in PBS and incubated with the tissue for 60 min in the dark. The tissue was washed with PBS, mounted with anti-fade medium, and observed under a fluorescence microscope.

### 2.13. Immunofluorescence

Cell: H9c2 cells were fixed with 4% paraformaldehyde at room temperature for 15 min and then infiltrated with 0.5% Triton X-100 (GC204003, Servicebio, Wuhan, China) for 15 min. H9c2 cells were blocked with 5% bovine serum albumin at room temperature for 1 h, incubated with p21 (A1483, Abclonal, Wuhan, China), p53 (10442–1–AP, Proteintech, Wuhan, China) and γ-H2AX (C2036S, Beyotime, Shanghai, China) primary antibodyovernight at 4 °C, incubated with an Alexa Fluor^®^ 488 AffiniPure™ Goat Anti-Rabbit IgG (H+L) (112–545–003, Jackson immunoResearch, West Grove, PA, USA), Multi-rAb^®^ CoraLite^®^ Plus 488-Goat Anti-Mouse Recombinant Secondary Antibody (H+L) (RGAM002, Proteintech, Wuhan, China) and Multi-rAb^®^ CoraLite^®^ Plus 594-Goat Anti-Rabbit Recombinant Secondary Antibody (H+L) (RGAR004, Proteintech, Wuhan, China) for 1 h, and then incubated with DAPI (G1012, Servicebio, Wuhan, China) for 10 min. The cells were washed three times with PBS for 5 min between each step.

Tissue: Paraffin sections were deparaffinized to water, followed by antigen retrieval using citrate buffer (pH 6.0) under high-pressure conditions. Subsequently, endogenous peroxidase activity was blocked with 3% hydrogen peroxide, and non-specific binding sites were blocked with serum. The sections were incubated with a specific primary antibody at 4 °C overnight, followed by incubation with an HRP-labeled secondary antibody at 37 °C for 1 h after returning to room temperature. A tyramine signal amplification (TSA) system was employed to enhance the fluorescent signal. For multiplex immunofluorescence staining, the above steps of primary antibody, secondary antibody, and TSA incubation were repeated for subsequent targets after removing the previous antibody complex via microwave antigen retrieval. Finally, the nuclei were counterstained with DAPI, and the sections were mounted for observation under a fluorescence microscope.

### 2.14. Histology Examination

Heart tissues were fixed in 4% paraformaldehyde and embedded in paraffin. Then, the paraffin blocks were cut into 4 μm thick slices and stained with a hematoxylin and eosin (HE) kit (G1121, Solarbio, Beijing, China). HE-stained sections were used for pathological examination.

### 2.15. RNA Isolation, Library Preparation and Analysis

Total RNA was isolated from the samples with Trizol reagent (Vazyme, Nanjing, China) in strict accordance with the manufacturer’s standard operating procedures. The purity and concentration of the extracted RNA were determined utilizing a NanoDrop 2000 spectrophotometer (Thermo Scientific, Waltham, MA, USA), while RNA integrity was further evaluated via an Agilent 2100 Bioanalyzer (Agilent Technologies, Santa Clara, CA, USA). Subsequent to the quality assessment, RNA libraries were prepared by following the protocols provided with the VAHTS Universal V10 RNA-seq Library Prep Kit (Premixed Version). Finally, the entire process of transcriptome sequencing and data analysis was entrusted to OE Biotech Co., Ltd. (Shanghai, China).

### 2.16. Superoxide Dismutase (SOD) Activity Assay

SOD levels were determined using a SOD activity assay kit (BC5165, Solarbio, Beijing, China). Harvested cells were washed twice with PBS. For 1 × 10^6^ cells, 0.5 mL of ice-cold SOD extraction buffer containing protease inhibitors was added, and cells were lysed on ice by ultrasonication (200 W, 3 s pulses, 10 s intervals, 30 cycles). The lysate was centrifuged at 12,000× *g* for 10 min at 4 °C, and the supernatant was collected and kept on ice for later analysis. Frozen myocardial tissues (20–30 mg) were mechanically homogenized on ice in ice-cold extraction buffer at a ratio of 1:10 (*w*/*v*). The homogenate was centrifuged at 12,000× *g* for 10 min at 4 °C, and the supernatant was harvested for subsequent assays. Protein concentration was determined using a BCA protein assay kit to normalize SOD activity. All procedures were performed strictly according to the manufacturer’s instructions.

### 2.17. Detection of Malondialdehyde (MDA) Levels

MDA levels were measured using a commercial MDA assay kit (BC0025, Solarbio, Beijing, China). Sample preparation was performed as described in Section 2.16. The remaining steps were conducted following the kit protocol.

### 2.18. Proliferation Assay (EdU)

Cell proliferation was evaluated using the EdU incorporation assay (G1601, Servicebio, Wuhan, China). In brief, a 2× EdU working solution was prepared by adding 2 μL of 10 mM EdU stock solution to 1 mL of pre-warmed complete medium. Cells were incubated with an equal volume of pre-warmed 2× EdU working solution (replacing half of the original culture medium) for 2 h. After one rinse with PBS, cells were fixed with fixative at room temperature for 15 min and washed twice with PBS (3 min each wash). Cells were then permeabilized with 0.2% Triton X-100 for 15 min at room temperature, followed by two washes with PBS (3 min each). Following PBS removal, click reaction buffer was added and incubated in the dark for 30 min at room temperature, after which cells were washed twice with PBS (3 min each). Nuclei were counterstained with Hoechst 33342 (1:1000 dilution in PBS) for 5 min in the dark, and then washed twice with PBS (3 min each). Images were acquired under a fluorescence microscope from randomly selected fields. The percentage of EdU-positive cells was calculated as (EdU-positive cells/total cells) × 100%.

### 2.19. Grip Strength Test

Prior to formal testing, mice were acclimated to the testing room for 30 min daily over three consecutive days and trained to grasp the grid of a grip strength meter with gentle tail pulling (2–3 sessions/day, 1-min intervals). For the formal test, each mouse was held by the tail, allowing its forelimbs to naturally grasp the grid while the body remained horizontal. The tail was then pulled backward at a constant speed until the forelimbs released the grid, and the peak force was recorded. This procedure was repeated three times per mouse with at least 30-s intervals to avoid fatigue, and the average value was taken as the final grip strength.

### 2.20. Animal Inclusion/Exclusion Criteria and Sample Selection

The inclusion criteria required animals to be in good health, well acclimated to the environment, and in a stable condition. Initially, 5 animals were allocated per group. Animals were excluded if their body weight decreased by more than 20% during the experimental period. No animals were excluded during the experimental period.

All animals from each group (*n* = 5) underwent comprehensive assessment of aging-related phenotypes, including body weight, heart weight, echocardiography, and grip strength. For molecular biology experiments involving cardiac tissue, the number of samples was limited by the size of the heart tissue. Therefore, three mice per group were randomly selected for all such analyses, including qRT-PCR, Western blot, WGA staining, HE staining, and IF staining. For qRT-PCR, sample selection was additionally based on predefined RNA quality control criteria, with only samples meeting A260/A280 ratios between 1.8 and 2.1, and A260/A230 ratios > 2.0 used. For Western blot analysis, three representative samples per group were randomly selected.

Sample sizes for each analysis are clearly indicated in the corresponding figure legends.

### 2.21. Statistical Analysis

Prior to statistical analyses, the normality of the data and the homogeneity of variances were assessed using Levene’s test, respectively. The data were processed with GraphPad Prism software 10 (GraphPad Software Inc., San Diego, CA, USA). The results are presented as the mean ± SEM and were compared between the two groups by using the *t* test. To compare more than two groups, one-way ANOVAs were conducted. Differences were considered statistically significant at *p* < 0.05. To minimize bias, a blinded design was implemented where feasible. Group allocation was performed by an independent researcher not involved in subsequent experiments. During the conducting of the experiment, the investigator administering daily D-gal injections and PCP treatment was necessarily aware of group allocation due to the need for different treatment regimens. However, all outcome assessments were performed under blinded conditions. For echocardiography and histological analysis (including WGA, HE, and immunofluorescence), the operators and analysts were unaware of group allocation. For Western blot analysis, while the individual responsible for sample loading was aware of group allocation to ensure correct loading order, subsequent image quantification was performed by a researcher blinded to group information using coded filenames. Data analysis for all other parameters was also conducted by researchers blinded to group allocation. This partial blinding strategy ensured that potential bias was minimized during critical stages of data interpretation while acknowledging practical technical constraints.

## 3. Results

### 3.1. Characteristic Assessment of Polygonatum cyrtonema Polysaccharides

**Figure 1:**
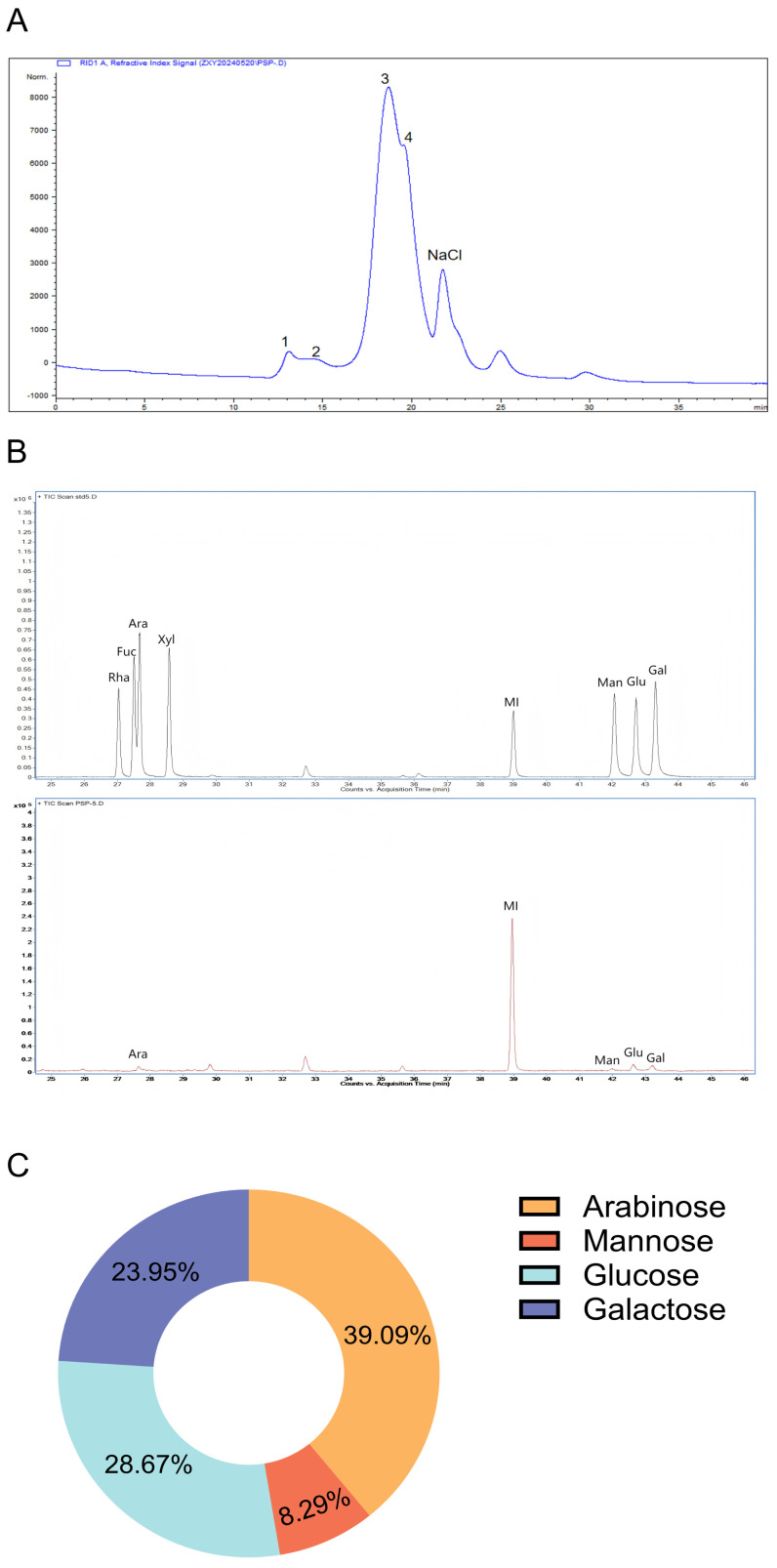
Chemical characterization of PCPs. (**A**) Molecular weight distribution. (**B**) Monosaccharide composition. (**C**) Proportional composition of monosaccharides in PCPs.

The total carbohydrate content of PCPs was 60.875% as determined via the phenol-sulfuric acid approach. The molecular weight distribution of PCPs was rigorously assessed using HPGPC. As depicted in Figure 1A, the HPGPC chromatogram of PCPs comprised four obvious peaks, from 5 kD to 776 kD. GC analysis revealed that PCPs were composed of four peaks that correspond to four types of monosaccharides: arabinose, mannose, glucose, and galactose in a 39.09:8.29:28.67:23.95 ratio (Figure 1B,C). This suggested that PCPs were primarily composed of arabinose and glucose.

### 3.2. PCPs Alleviated D-Gal-Induced Cellular Senescence in H9c2 Cardiomyocytes

**Figure 2:**
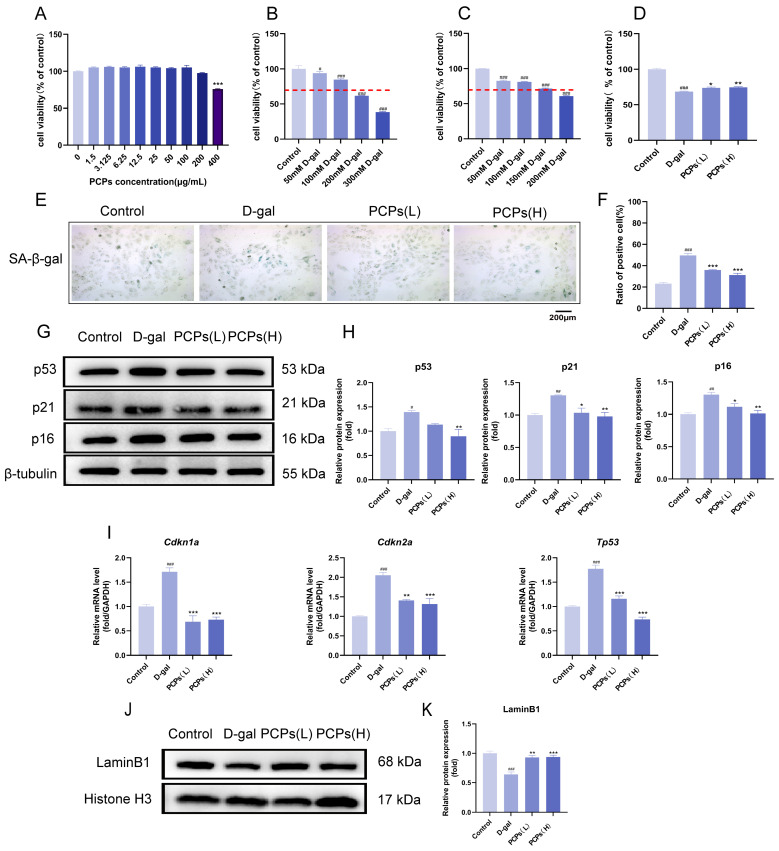
PCPs ameliorate D-gal-induced cardiomyocyte senescence. (**A**,**B**) Screening of the optimal D-gal concentration for model establishment. (**C**) Cytotoxicity assessment of PCPs. (**D**) Effect of PCPs on the proliferation inhibition of senescent H9c2 cardiomyocytes measured by CCK-8 assay. (**E**,**F**) Senescence assessment in H9c2 cardiomyocytes by SA-β-gal staining following PCP treatment. (**G**,**H**) Western blot analysis of senescence-associated protein expression. (**I**) mRNA expression levels of senescence-related genes in H9c2 cardiomyocytes. (**J**,**K**) Western blot analysis of senescence-associated nuclear protein expression. The data are shown as the mean ± SEM, *n* = 3/group; ^#^ *p* < 0.05 versus the control group; * *p* < 0.05 versus the D-gal group. * *p* < 0.05, ** *p* < 0.01, *** *p* < 0.001, ^#^ *p* < 0.05, ^##^ *p* < 0.01, ^###^ *p* < 0.001.

To exclude potential interference of PCPs with the basal growth of H9c2 cardiomyocytes, cell viability was first assessed using the CCK-8 assay (Figure 2A). Based on the results, two concentrations of PCPs (100 μg/mL and 200 μg/mL) were selected for subsequent experiments. Meanwhile, the optimal concentration of D-gal for establishing the senescence model was determined using the same method, with a target viability reduction to approximately 70% of the control group. Ultimately, 150 mM D-gal was chosen to induce cellular senescence (Figure 2B,C).

As a hallmark of cellular senescence, cell cycle arrest and proliferation inhibition can be directly reflected by cell viability. The results demonstrated that compared with the control group, cell viability in the D-gal-treated group was significantly decreased, indicating suppressed proliferation of cardiomyocytes. In contrast, treatment with different concentrations of PCPs resulted in a dose-dependent recovery of cell viability, suggesting that PCPs effectively ameliorated D-gal-induced proliferation inhibition (Figure 2D).

β-galactosidase is a classic biochemical marker of cellular senescence, with its activity markedly elevated in senescent cells. SA-β-gal staining revealed that the percentage of SA-β-gal-positive cells was significantly higher in the D-gal-induced model group than in the control group. In contrast, PCP treatment notably reduced the proportion of SA-β-gal-positive cells (Figure 2E,F).

p53, p16, and p21 are core molecules within the regulatory network of cellular senescence, collaboratively driving the senescence process [32]. Changes in their expression are first detectable at the transcriptional level before subsequently affecting protein expression. We initially examined the protein expression levels of p53, p16, and p21 in cardiomyocytes by Western blot analysis (Figure 2G,H). The results revealed that compared with the normal control group, the D-galactose-induced model group exhibited significantly increased expression of all three proteins, indicating the establishment of a senescent state. Subsequently, we quantified the mRNA expression levels of p53 (*Tp53*), p16 (*Cdkn2a*), and p21 (*Cdkn1a*) using qPCR. The analysis demonstrated that the mRNA expression of these genes was also significantly upregulated in the model group, consistent with the protein-level findings, confirming enhanced transcriptional activity of senescence-associated genes (Figure 2I). Following PCP intervention, both mRNA and protein expression levels of these markers were downregulated in a dose-dependent manner. Collectively, this coherent evidence from translational to transcriptional levels robustly demonstrates the anti-senescent effects of PCPs on cardiomyocytes.

LaminB1, a critical structural protein of the nuclear lamina, shows markedly decreased expression during cellular senescence, leading to nuclear membrane disruption, abnormal nuclear morphology, and impaired chromatin stability and gene expression regulation [33]. Analysis of LaminB1 protein expression in cardiomyocytes (Figure 2J,K) revealed that its level was significantly lower in the D-gal-induced model group than in the normal control group. In contrast, PCP treatment notably restored LaminB1 expression, supporting the conclusion that PCPs exert anti-senescent effects by helping to maintain nuclear membrane integrity.

Aging is typically accompanied by the inhibition of cell proliferation and alterations in the expression of specific proteins. To evaluate the effect of PCPs on D-gal-induced cardiomyocyte senescence, we analyzed both cellular proliferative capacity and key senescenc-related signaling pathways using EdU proliferation assays and p53/p21 immunofluorescence staining, respectively.

**Figure 3:**
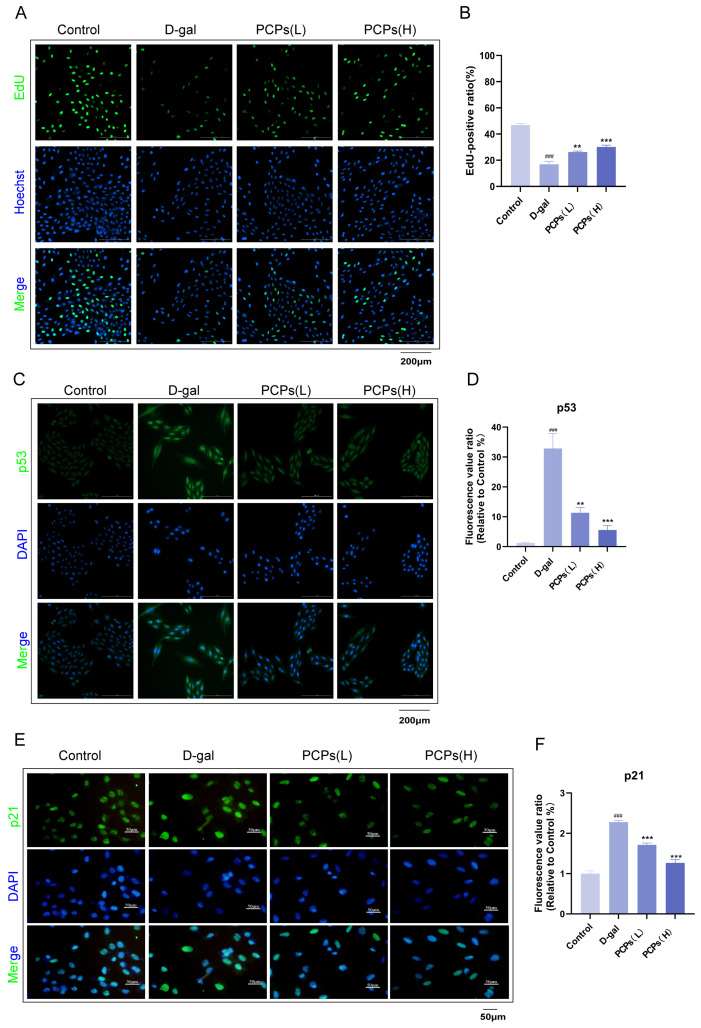
Immunofluorescence detection of EdU, p21 and p53 in D-gal-induced aging cells with PCPs intervention. (**A**,**B**) EdU staining. Green: EdU; blue: Hoechst 33342. Scale bar, 200 μm. (**C**,**D**) Immunofluorescence staining images (green: p53; blue: DAPI) and quantitative analysis of p53 expression, a senescence marker. Scale bar, 200 μm. (**E**,**F**) Immunofluorescence staining images (green: p21; blue: DAPI) and quantitative analysis of p21 expression, a senescence marker. Scale bar, 50 μm. The data are shown as the mean ± SEM, *n* = 3/group; ^###^ *p* < 0.001 versus the control group; ** *p* < 0.01 versus the D-gal group. ** *p* < 0.01, *** *p* < 0.001.

The EdU assay results showed that D-gal treatment significantly reduced the EdU-positive rate in H9c2 cells, indicating a marked suppression of proliferative activity. In contrast, intervention with PCPs led to a significant recovery of the EdU-positive rate (Figure 3A,B), demonstrating that PCPs can effectively reverse the proliferation arrest induced by D-gal.

In the p53/p21 immunofluorescence analysis, enhanced fluorescence signals of both p53 and its downstream target p21 were observed after D-gal induction, suggesting activation of the p53/p21 pathway. Following PCP treatment, the fluorescence intensities of both proteins were markedly attenuated (Figure 3C–F), indicating that PCPs can suppress the activation of this senescence-associated signaling pathway.

In summary, the EdU experiment functionally confirms the role of PCPs in promoting cell proliferation, while the p53/p21 immunofluorescence data reveal, at the molecular level, the ability of PCPs to inhibit the activation of a key senescence pathway. These results are consistent with previous protein and mRNA-level findings and collectively demonstrate that PCPs exert anti-senescence effects in cardiomyocytes by modulating the p53/p21 pathway and alleviating cell cycle arrest.

### 3.3. PCPs Ameliorate D-Gal-Induced ROS Accumulation and Mitochondrial Dysfunction in Cardiomyocytes

**Figure 4:**
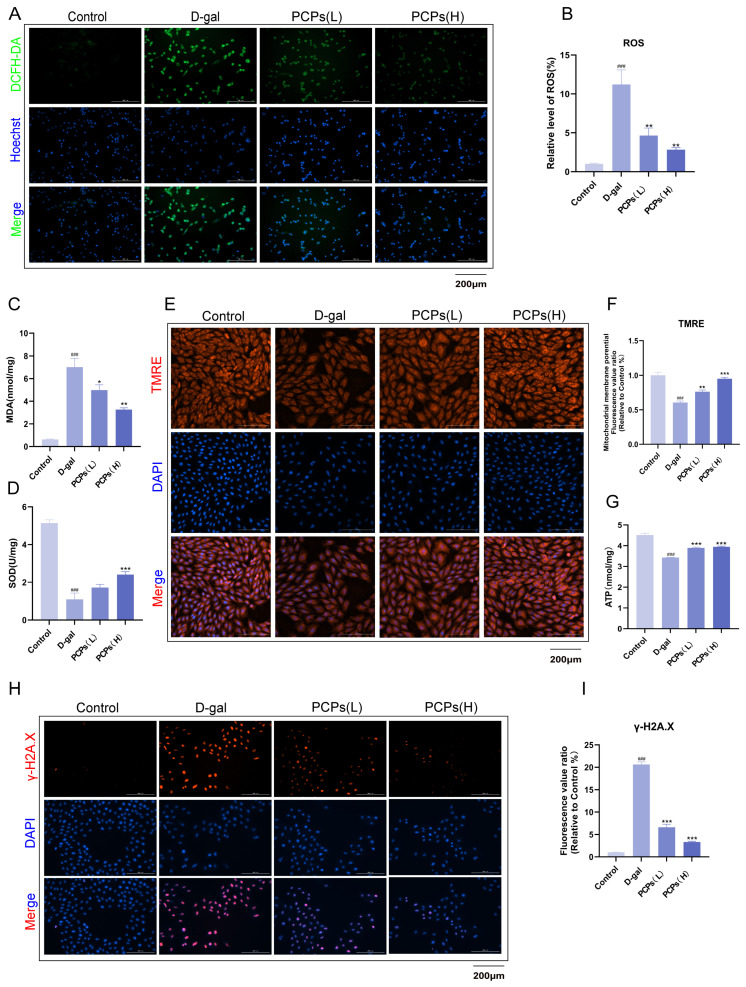
PCPs ameliorate D-gal-induced mitochondrial dysfunction in H9c2 cardiomyocytes. (**A**) Intracellular ROS levels detected by DCFH-DA fluorescent probe. Green: DCFH-DA; blue: Hoechst 33342. Scale bar, 200 μm. (**B**) Quantitative analysis of ROS fluorescence intensity. (**C**) MDA content in each group. (**D**) SOD activity levels across experimental groups. (**E**,**F**) Changes in mitochondrial membrane potential (ΔΨm) measured by TMRE fluorescent probe. Red: TMRE; blue: DAPI. Scale bar, 200 μm. (**G**) ATP content in H9c2 cells. (**H**,**I**) Immunofluorescence analysis of γ-H2AX foci (marker of DNA double-strand breaks). Red: γ-H2AX; blue: DAPI. Scale bar, 200 μm. The data are shown as the mean ± SEM, *n* = 3/group; * *p*< 0.05 versus the D-gal group. * *p* < 0.05, ** *p* < 0.01, *** *p* < 0.001, ^###^ *p* < 0.001.

During its intracellular metabolism, D-gal induces excessive generation of ROS by promoting the accumulation of advanced glycation end products (AGEs) and activating NADPH oxidase, thereby disrupting the cellular redox balance [34]. Accordingly, we first detected intracellular ROS levels. The results showed that compared with the normal control group, the D-gal-treated group exhibited a significant increase in ROS fluorescence intensity, indicating that D-gal induces substantial ROS production in cardiomyocytes and triggers oxidative stress. In contrast, intervention with PCPs resulted in a dose-dependent reduction in ROS fluorescence intensity (Figure 4A,B), confirming that PCPs effectively scavenge excess intracellular ROS and alleviate oxidative stress damage. SOD and MDA are two key biomarkers for assessing oxidative stress levels: SOD is a core antioxidant enzyme responsible for scavenging free radicals, while MDA is a stable end product of lipid peroxidation damage. Experimental results showed that in D-gal-induced senescent cardiomyocytes, SOD activity was significantly decreased, while MDA content was markedly increased, indicating severe oxidative damage. Following intervention with PCPs, SOD activity was restored, and MDA production was significantly reduced (Figure 4C,D). These findings demonstrate that PCPs can effectively delay cellular senescence by enhancing the antioxidant defense system and alleviating lipid peroxidation.

Given that excessive ROS impairs mitochondrial function, we further examined changes in MMP using the TMRE fluorescent probe, which specifically labels MMP (Figure 4E,F). In the control group, cells displayed strong and uniformly distributed TMRE fluorescence, indicating stable MMP and normal mitochondrial function. In contrast, the D-gal-induced model group showed a significant reduction in TMRE fluorescence intensity, suggesting a substantial decline in MMP and impaired mitochondrial function. PCPs intervention led to a concentration-dependent restoration of TMRE fluorescence. Since a decrease in MMP affects ATP synthesis, we also measured intracellular ATP levels. The observed changes in ATP content were fully consistent with the MMP results (Figure 4G). These findings indicate that PCPs can reduce ROS levels, thereby maintaining MMP stability and improving mitochondrial function in D-gal-injured cardiomyocytes.

Given that mitochondrial dysfunction can subsequently induce DNA damage, we assessed double-strand breaks using an anti-γ-H2AX antibody combined with immunofluorescence (Figure 4H,I). Cells in the control group exhibited only very faint γ-H2AX fluorescence, indicating minimal DNA damage. In contrast, the D-gal-induced model group showed a significant increase in γ-H2AX fluorescence intensity, suggesting severe DNA damage. Treatment with PCPs resulted in a concentration-dependent reduction in γ-H2AX fluorescence signals. These results confirm that by preserving mitochondrial function, PCPs can mitigate D-gal-induced DNA damage in cardiomyocytes and help maintain genomic stability.

### 3.4. PCPs Delay D-Gal-Induced Cardiomyocytes Senescence by Inhibiting the p38 MAPK Pathway

**Figure 5:**
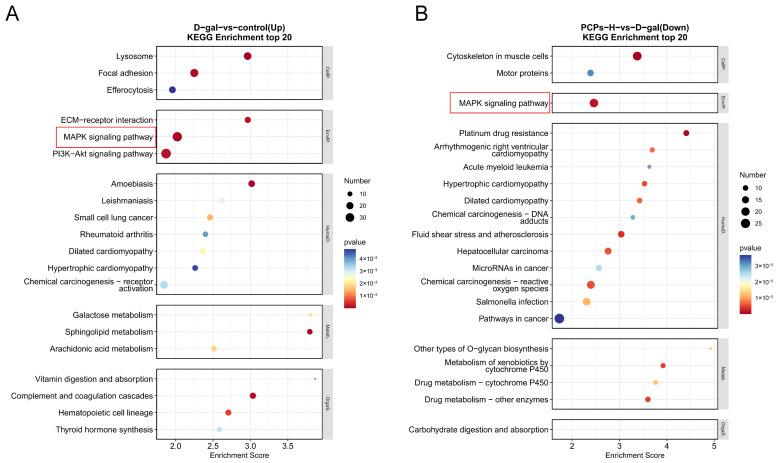
Transcriptome analysis of H9c2 cardiomyocytes in D-gal-induced aging model with PCP intervention. (**A**) Control vs. D-gal group. (**B**) PCPs H vs. D-gal group. The red frame indicates the common pathways screened in this study.

**Figure 6:**
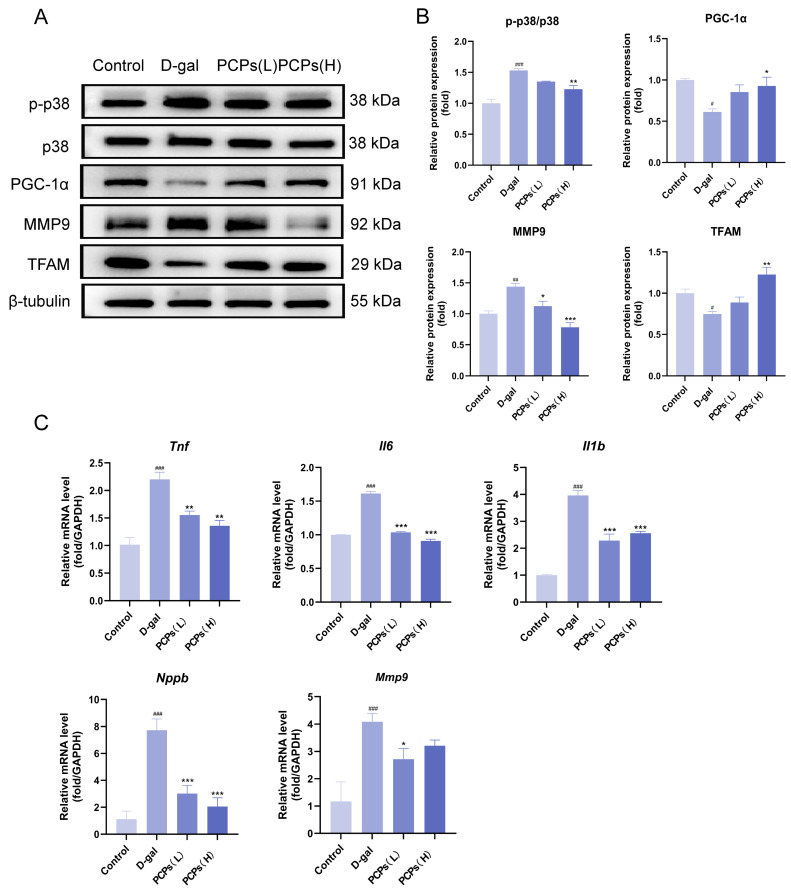
PCPs ameliorate D-gal-induced oxidative stress and chronic inflammation in H9c2 cardiomyocytes. (**A**,**B**) Western blot analysis of protein expression related to oxidative stress and inflammation. (**C**) mRNA expression levels of inflammation-related genes in H9c2 cells. The data are shown as the mean ± SEM, *n* = 3/group; ^#^ *p* < 0.05 versus the control group; * *p* < 0.05 versus the D-gal group. * *p* < 0.05, ** *p* < 0.01, *** *p* < 0.001, ^#^ *p* < 0.05, ^##^ *p* < 0.01, ^###^ *p* < 0.001.

To explore the specific molecular mechanisms underlying the anti-cardiac aging effects of PCPs, we performed transcriptome sequencing analysis on H9c2 cardiomyocytes. Transcriptomic data revealed that in the D-gal-induced cellular aging model, the global expression of genes related to the mitogen-activated protein kinase (MAPK) signaling pathway was significantly upregulated relative to the control group (Figure 5A). Notably, PCPs intervention led to a marked downregulation in the expression of these MAPK pathway-associated genes (Figure 5B). The MAPK signaling cascade consists of three major subfamilies, namely extracellular regulated protein kinases (ERK), c-Jun N-terminal kinases (JNK), and p38 MAPK. Based on previous literature reports, p38 MAPK—an important molecule closely associated with oxidative stress and cellular senescence—was selected for subsequent validation experiments. Our data confirmed that D-gal activates the p38 MAPK pathway through ROS accumulation induced by oxidative stress. On the one hand, activated p38 inhibits the expression of peroxisome proliferator-activated receptor gamma coactivator 1-alpha (PGC-1α) via phosphorylation, which in turn reduces the synthesis of its downstream effector transcription factor A, mitochondrial (TFAM). This impairment ultimately disrupts mitochondrial DNA replication and respiratory chain function. On the other hand, activated p38 also induces the overexpression of matrix metalloproteinase 9 (MMP9). MMP9 degrades the extracellular matrix, promotes myocardial structural remodeling, and further compromises antioxidant capacity, thereby forming a vicious cycle of oxidative damage. Western blot analysis demonstrated that PCPs could inhibit p38 phosphorylation, upregulate the expression of the PGC-1α/TFAM axis, and simultaneously reduce MMP9 levels, thereby alleviating myocardial damage and delaying cardiomyocyte senescence (Figure 6A,B).

To further elucidate the role of p38 activation in the senescence-associated secretory phenotype (SASP) and myocardial remodeling, we detected the expression of its downstream key effector molecules using qPCR. The results demonstrated that D-gal induction led to a marked upregulation in the mRNA levels of pro-inflammatory factorsIL-6 (*Il6*), TNF-α (*Tnf*), IL-1β (*Il1b*), B-type natriuretic peptide (*Nppb*), and MMP9(*Mmp9*) in cardiomyocytes. In contrast, intervention with PCPs effectively abrogated the elevated expression of these molecules. Collectively, these findings indicate that PCPs may mitigate inflammatory responses, ameliorate myocardial stress indicators, and inhibit extracellular matrix degradation by suppressing the p38 signaling pathway, thereby attenuating the SASP (Figure 6C).

### 3.5. PCPs Ameliorate D-Gal-Induced Aging Phenotypes and Cardiac Senescence in Mice

**Figure 7:**
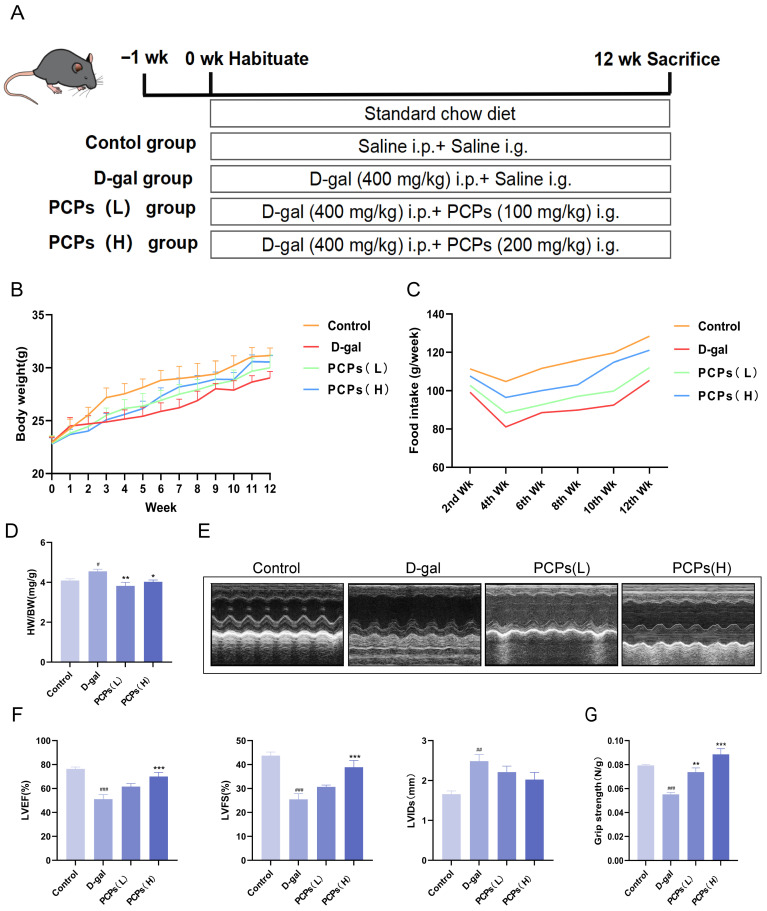
PCPs ameliorate D-gal-induced aging phenotypes and cardiac dysfunction in mice. (**A**) Schematic diagram of the experimental design. (**B**) Body weight changes during the experimental period. (**C**) Changes in food intake. (**D**) Heart weight to body weight ratio (HW/BW) across groups. (**E**) Representative echocardiographic images. (**F**) Quantitative analysis of cardiac function parameters. (**G**) Forelimb grip strength test results. The data are shown as the mean ± SEM, *n* = 5/group; ^#^ *p* < 0.05 versus the control group; * *p* < 0.05 versus the D-gal group. * *p* < 0.05, ** *p* < 0.01, *** *p* < 0.001, ^#^ *p* < 0.05, ^##^ *p* < 0.01, ^###^ *p* < 0.001.

An aging mouse model was established by continuous intraperitoneal injection of D-gal (400 mg/kg/day) for 12 weeks, with experimental design adapted from reference [35] and modified based on preliminary results. During modeling, the high- and low-dose PCP groups received daily oral administration of PCPs at 100 and 200 mg/kg/day, respectively (Figure 7A). Body weight was measured weekly, and food intake was recorded biweekly throughout the experimental period. The results showed that compared with the control group, D-gal-induced aging mice exhibited slower body weight gain and significantly reduced food intake (Figure 7B,C), demonstrating typical aging characteristics. Intervention with PCPs improved both body weight and food intake in the low-and high-dose groups.

Cardiac mass-to-body mass ratio and echocardiographic assessments were performed to evaluate cardiac morphological and functional changes associated with aging. The D-gal-induced aging model exhibited a markedly elevated cardiac mass-to-body mass ratio relative to the control group (Figure 7D), while PCP intervention significantly reversed this abnormal elevation. Consistent with the morphological changes, echocardiographic results revealed that D-gal stimulation led to obvious cardiac dysfunction in experimental subjects, as characterized by decreased left ventricular ejection fraction (LVEF) and left ventricular fractional shortening (LVFS), while left ventricular internal diameter at end-systole (LVID) was increased (Figure 7E,F), indicating impaired ventricular systolic function.

Concurrently, we assessed the forelimb grip strength of mice in each group and found that the D-gal group displayed a significant decline in grip strength, which was subsequently ameliorated by PCP treatment (Figure 7G). As grip strength is a recognized phenotypic indicator of aging, these findings collectively demonstrate that PCPs can alleviate systemic aging phenotypes induced by D-gal.

**Figure 8:**
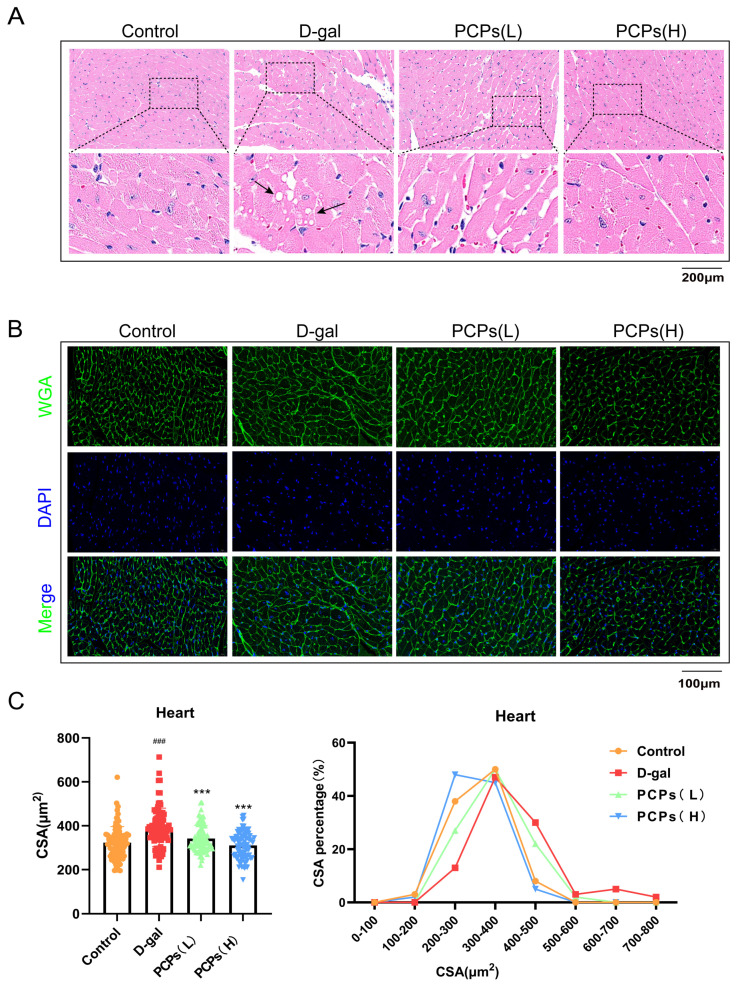
HE and WGA staining of D-gal-induced aging cardiac tissues. (**A**) HE staining of myocardial tissue. Scale bar, 200 μm. (**B**,**C**) WGA staining of myocardial tissue and quantitative analysis of cardiomyocyte cross-sectional area. Green: WGA; blue: DAPI. Scale bar, 100 μm. The data are shown as the mean ± SEM, *n* = 3/group; ^###^ *p* < 0.001 versus the control group; *** *p* < 0.001 versus the D-gal group.

Pathological analysis of myocardial tissue sections showed vacuolar degeneration of cardiomyocytes in the D-gal group. (Figure 8A). In contrast, the PCP intervention group exhibited markedly alleviated structural disorganization, with cellular morphology approaching normality. Cardiac aging often triggers myocardial compensation, where structural compensation primarily manifests as cardiomyocyte hypertrophy. This study evaluated the cardiomyocyte cross-sectional area (Figure 8B,C). The results demonstrated that D-gal-induced aging mice exhibited a significant increase in cardiomyocyte cross-sectional area and elevated HW/BW ratio, indicating pronounced characteristics of cardiomyocyte hypertrophy. Notably, PCP intervention resulted in a dose-dependent amelioration of these parameters: the high-dose PCP group showed a marked reduction in cardiomyocyte cross-sectional area and a decreased HW/BW ratio compared to the D-gal group. Despite the significant differences in body weight observed among experimental groups, the HW/BW ratio was used as an estimative parameter to assess cardiac hypertrophy. However, absolute heart weight and left ventricular mass data showed similar trends but did not reach statistical significance (Supplementary Figure S1).

### 3.6. PCPs Ameliorate Cardiac Aging in Mice by Modulating Oxidative Stress, Inflammatory Factors, and Senescence-Associated Proteins and Genes

**Figure 9:**
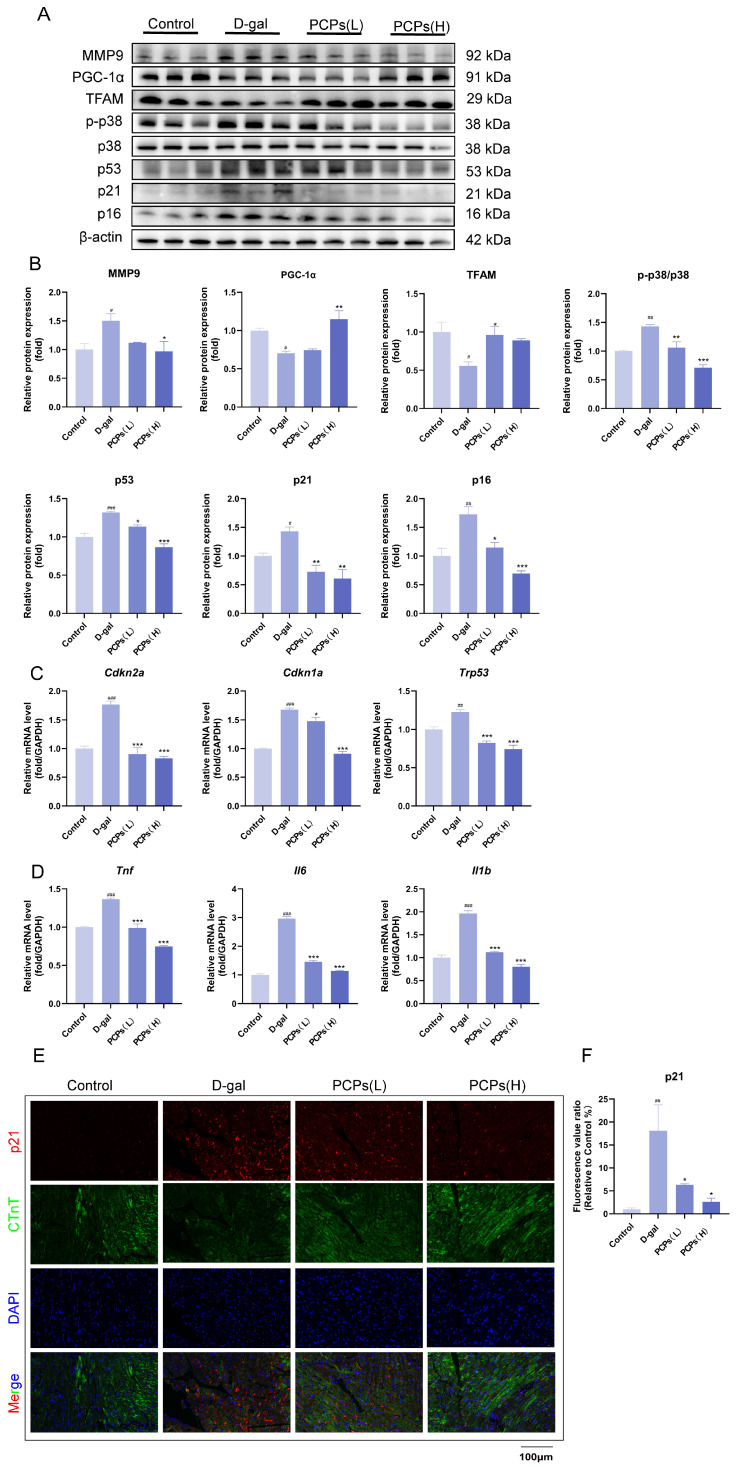
PCPs alleviate D-galactose-induced cardiac aging, oxidative stress, and chronic inflammation in mice. (**A**,**B**) Western blot analysis of senescence, oxidative stress, and inflammation-associated protein expression in myocardial tissues. (**C**,**D**) mRNA expression levels of senescence- and inflammation-related genes in cardiac tissue. (**E**,**F**) Immunofluorescence staining of cardiac tissue, Green: CTnT; red:p21; blue: DAPI. Scale bar, 100 μm. The data are shown as the mean ± SEM, *n* = 3/group; ^#^ *p* < 0.05 versus the control group; * *p* < 0.05 versus the D-gal group. * *p* < 0.05, ** *p* < 0.01, *** *p* < 0.001, ^#^ *p* < 0.05, ^##^ *p* < 0.01, ^###^ *p* < 0.001.

In the cardiac tissue of the aging D-gal group mice, compared with the control group, the phosphorylation level of p38 was significantly increased. Concurrently, the protein expression of PGC-1α, a key regulator of mitochondrial function, was significantly reduced, accompanied by a synchronous downregulation of its downstream target molecule TFAM. This suggests that p38 activation may exacerbate mitochondrial dysfunction by inhibiting the PGC-1α pathway. Further examination of senescence-associated pathway molecules revealed that the D-gal group exhibited significantly elevated protein and mRNA expression levels of p53 (*Trp53*), p21 (*Cdkn1a*), and p16 (*Cdkn2a*). In contrast, both the low- and high-dose PCP groups showed a marked reduction in their expression, indicating that PCPs can ameliorate cardiac aging in mice (Figure 9A–C). Moreover, p38 phosphorylation can activate downstream inflammatory factors, while mitochondrial dysfunction also promotes the release of these mediators. Therefore, we quantified the mRNA expression levels of IL-6 (*Il6*), TNF-α (*Tnf*), and IL-1β (*Il1b*) in cardiac tissue. The results revealed a significant increase in inflammatory markers in the D-gal group, which was markedly attenuated by treatment with PCPs (Figure 9D). These findings indicate that PCPs possess notable anti-inflammatory properties, likely contributing to their overall anti-aging effects. In addition, we performed immunofluorescence staining on cardiac tissues. The results demonstrated a significant increase in p21 fluorescence intensity in the D-gal group but a decreasing trend in the PCP group, which is consistent with the in vitro results (Figure 9E,F).

## 4. Discussion

This study systematically investigated the protective effects and underlying mechanisms of PCPs—the primary active component of the medicinal and edible plant *Polygonatum cyrtonema Hua*.—on cardiac aging. By integrating in vivo animal experiments with in vitro cellular studies, we aimed to establish a comprehensive mechanistic link between whole-organism observations and direct cellular actions. The results demonstrated that PCP intervention significantly improved cardiac function in aging model mice and exerted positive regulatory effects on the p53 pathway, PGC-1α/TFAM signaling, and mitochondrial function. These findings suggest that this bioactive substance, derived from a medicinal and edible source, holds promise as a potential candidate for delaying cardiac aging.

Cardiac aging represents one of the most prominent degenerative organ changes during organismal aging. Its key pathological features include a reduction in cardiomyocyte number accompanied by cellular hypertrophy, increased myocardial fibrosis, and declined ventricular diastolic and systolic function [36]. Echocardiographic analysis in this study revealed that compared with the control group, the aging model mice exhibited significantly decreased LVEF and LVFS, along with a markedly increased LVID, indicating substantial impairment of cardiac systolic function due to aging. Further histopathological examination showed disordered arrangement of myocardial fibers and cardiomyocyte hypertrophy in the model group, both of which were significantly ameliorated after intervention with PCPs. These results suggest that PCPs may protect cardiac function by maintaining the structural integrity of cardiomyocyte.

p53, a critical tumor suppressor gene and senescence regulator, plays a key role in cardiac aging [37]. Abnormal activation of the p53 pathway in senescent cardiomyocyte can induce cell cycle arrest and cellular senescence by upregulating downstream targets such as p21 [38], contributing to cardiac functional decline. Our experimental results demonstrated that the mRNA and protein expression levels of p53, p21, and p16 were significantly elevated in myocardial tissue of aging model mice, and intervention with PCPs markedly downregulated their expression. Consistent with these in vivo observations, parallel in vitro experiments using D-gal-induced senescent H9c2 cardiomyocytes revealed that PCPs treatment dose-dependently suppressed the upregulation of p53, p21, and p16. The inhibitory effect of PCPs on this pathway thus represents a key mechanism through which they alleviate senescence-associated phenotypes and improve cardiac function.

Mitochondrial dysfunction is a central hallmark of cardiac aging [39], and the PGC-1α/TFAM signaling pathway plays a pivotal regulatory role in maintaining mitochondrial function. PGC-1α, a master transcriptional coactivator, directly regulates the expression of TFAM, thereby influencing mitochondrial DNA replication, transcription, and translation, which is essential for preserving mitochondrial structural and functional integrity [40]. This study found that the expression levels of PGC-1α and TFAM were significantly reduced in the aging cardiac, accompanied by a marked decrease in MMP and ATP production, indicating mitochondrial dysfunction. Cardiomyocytes, which are highly dependent on mitochondrial energy supply [41], are particularly susceptible to these changes. The reduction in MMP reflects impaired electron transport chain function, directly compromising ATP synthesis and contractile performance. Intervention with PCPs significantly upregulated the expression of PGC-1α and TFAM, enhanced mitochondrial membrane potential, and increased ATP content. These results suggest that PCPs improve mitochondrial function by activating the PGC-1α/TFAM pathway, thereby supplying sufficient energy to cardiomyocytes—a key mechanism that may underlie its beneficial effects on cardiac contractile function.

A key unresolved question is the relationship between the p53 and PGC-1α pathways. Given that excessive ROS generation is a common upstream event in D-gal-induced aging, it is plausible that PCPs may act on this shared trigger, simultaneously alleviating p53 activation and restoring PGC-1α-mediated mitochondrial function, rather than targeting these pathways independently.

Chronic inflammation and oxidative stress are key hallmarks of the aging process and are closely associated with cardiac aging [42]. Although this study did not directly examine the related signaling pathways, the observed improvement in mitochondrial function may indirectly influence these processes. Mitochondria are the primary source of intracellular ROS, and mitochondrial dysfunction leads to excessive ROS production, triggering oxidative stress damage [43]. Concurrently, signaling molecules released from damaged mitochondria can activate inflammatory responses, creating a vicious cycle [44]. PCPs treatment enhanced antioxidant capacity consistently across both in vitro and in vivo models. In cultured cardiomyocytes, PCPs elevated SOD activity and decreased MDA content—effects that were similarly observed in myocardial tissue of aging mice. By bolstering antioxidant defenses, PCPs may attenuate ROS generation and subsequently suppress senescence-associated inflammatory mediators. This is consistent with the observed downregulation of SASP-related inflammatory factors, indicating that the anti-inflammatory effects of PCPs in vivo may derive, at least in part, from improved redox homeostasis at the cellular level.

*Polygonati rhizoma*, a traditional Chinese medicine for nourishing yin, contains polysaccharides with diverse biological activities that have garnered significant attention in anti-aging research. Previous studies have shown that PCPs can delay organismal aging by improving energy metabolism, modulating gut microbiota, and inhibiting cellular senescence. However, research on its effects on cardiac aging and the underlying mechanisms remains limited. This study provides the first evidence that PCPs ameliorate cardiac aging through coordinated regulation of p53-mediated senescence and PGC-1α/TFAM-mediated mitochondrial function, improving mitochondrial function, enhancing mitochondrial membrane potential and ATP production. These actions collectively improve cardiac function and alleviate aging-related pathological changes in the myocardium, offering new experimental support for the anti-aging application of PCPs.

Compared with other anti-aging agents, PCPs offer distinct advantages. For instance, although resveratrol can delay cardiac aging by activating the SIRT1 pathway, its low bioavailability and potential safety concerns associated with long-term use remain contentious [45]. In contrast, *Polygonatum cyrtonema*, as a medicinal and edible resource, demonstrates a high safety profile. A 10-week toxicological study reported no significant adverse reactions in mice even when administered *Polygonatum* extract at doses exceeding 2000 mg/kg [46], supporting its suitability for long-term intervention. Furthermore, our study found that PCPs exerted significant cardioprotective effects at a relatively low dose (100 mg/kg), highlighting its promising potential for practical applications.

This study has several limitations. First, the experiments utilized a D-gal-induced accelerated aging model, which differs from natural aging processes. Future studies should incorporate natural aging models to validate the findings and enhance their robustness. Second, while we observed that PCPs concurrently modulate p53 and PGC-1α/TFAM pathways, the precise interactive mechanisms between these pathways remain to be elucidated. Given that excessive ROS is a common upstream trigger in this aging model, future investigations should explore whether PCPs act primarily by alleviating oxidative stress, thereby simultaneously influencing both pathways, rather than targeting them independently. Third, while systolic function improved following PCP treatment, diastolic function—a critical indicator of cardiac aging—was not evaluated. This limitation stems from the absence of the Doppler module on the echocardiography system at the time of data acquisition, precluding assessment of parameters such as the ratio of early diastolic transmitral flow velocity to late diastolic transmitral flow velocity (E/A ratio), the ratio of early diastolic transmitral flow velocity to early diastolic mitral annular velocity (E/e’ ratio), and isovolumic relaxation time (IVRT). Future investigations should incorporate these diastolic measures using a system with full Doppler functionality. Fourth, while PCPs treatment concurrently reduced oxidative stress and improved mitochondrial function in both in vivo and in vitro models, the precise mechanism by which PCPs exert these effects remains to be fully elucidated. A key unresolved question is whether PCPs act primarily as direct ROS scavengers or whether they restore mitochondrial function, thereby reducing ROS generation as a secondary consequence. Our current experimental data, while demonstrating clear antioxidant and mitochondrial-protective effects, do not definitively distinguish between these two possibilities. Further experiments, including direct ROS scavenging assays and time-course analyses, are needed to clarify whether PCPs act primarily as direct ROS scavengers or indirectly via mitochondrial protection.

## 5. Conclusions

In summary, this study demonstrates that PCPs ameliorate cardiac function during aging through coordinated regulation of p53-mediated senescence and PGC-1α/TFAM-mediated mitochondrial function. This leads to improved mitochondrial function, restoration of MMP, increased ATP synthesis and enhanced antioxidant capacity. These findings not only provide new theoretical insights into the molecular mechanisms of cardiac aging but also establish an experimental foundation for developing PCPs against age-related cardiac dysfunction. With further research, PCPs hold promising potential for delaying cardiac aging and contributing to the prevention and treatment of cardiovascular diseases in the elderly.

## Data Availability

Dataset available on request from the authors.

## Supplementary Materials

The following supporting information can be downloaded at: https://www.mdpi.com/article/10.3390/nu18091390/s1, Table S1: Antibody reagent list; Table S2: Primers for RT-qPCR; Figure S1: Heart weight and left ventricular mass. (A) Heart weight. (B) Left ventricular mass. The data are shown as the mean ± SEM, *n* = 5/group.

## Abbreviations

The following abbreviations are used in this manuscript:

CSA	Cross-sectional area
D-gal	D-Galactose
DMEM	Dulbecco’s Modified Eagle Medium
FBS	Fetal bovine serum
GC	Gas chromatography
HE	Hematoxylin and eosin
MDA	Malondialdehyde
PBS	Phosphate-buffered saline
PVDF	Polyvinylidene difluoride
PCPs	*Polygonatum cyrtonema* polysaccharides
ROS	Reactive oxygen species
SOD	Superoxide dismutase
TMRE	Tetramethylrhodamine
