# Supplementary material for: Exploring the Therapeutic Effect of Polygonatum cyrtonema Polysaccharides in Reversing D-Galactose (D-Gal)-Mediated Cardiac Aging
Source: Nutrients. 2026 Apr 28;18(9):1390. doi: 10.3390/nu18091390 (PMC13165075; doi:10.3390/nu18091390)
Supplement: Supplementary file 1 [file nutrients-18-01390-s001.zip › Supplementary Table S1.pdf]

Table S1. Antibody reagent list

Antibodies	Dilution	Source	Identifier
β-Tubulin	1:1000	Abclonal	A12289
Histone H3	1:1000	Proteintech	17168-1-AP
p21	1:1000	Abclonal	A1843
p53	1:1000	Proteintech	10442-1-AP
p16	1:1000	Proteintech	28416-1-AP
LaminB1	1:1000	Abclonal	A1910
p38 MAPK	1:1000	Cell Signaling Technology	9212S
Phospho-p38 MAPK	1:1000	Cell Signaling Technology	4511T
MMP9	1:1000	Abcam	ab283575
PGC-1α	1:1000	Zenbio	R381615
TFAM	1:1000	Abclonal	A3173
beta Actin	1:5000	Zenbio	700068
Goat Anti-Rabbit IgG(H+L)HRP	1:20000	BioTNT	A20120A0704
Goat Anti-Mouse IgG(H+L)HRP	1:20000	BioTNT	A20120A0703
AffiniPure® Goat Anti-Rabbit IgG (H+L)	1:200	Jackson	111-545-003
Multi-rAb™ CoraLite® Plus 488-Goat Anti-Mouse Recombinant Secondary Antibody (H+L)	1:200	Proteintech	RGAM002
