# Supplementary material for: Exploring the Therapeutic Effect of Polygonatum cyrtonema Polysaccharides in Reversing D-Galactose (D-Gal)-Mediated Cardiac Aging
Source: Nutrients. 2026 Apr 28;18(9):1390. doi: 10.3390/nu18091390 (PMC13165075; doi:10.3390/nu18091390)
Supplement: Supplementary file 1 [file nutrients-18-01390-s001.zip › Supplementary Table S2.pdf]

Table S2. Primers for RT-qPCR.

Species	Primer	Sequence (5’ to 3’)
Rat	Mmp9 F	CTGAGGCCCCTACAGAGTCT
Rat	Mmp9 R	GGCAATAAGAAAGGGGCCCT
Rat	Nppb F	AGCTCTCAAAGGACCAAGGC
Rat	Nppb R	TCCGGTCTATCTTCTGCCCA
Rat	Tp53 R	GCAGCACAGGAACCTGGAACTG
Rat	Tp53 F	AGAAGGGACGGAAGATGACAGAGG
Rat	Cdkn2a F	ACAGGTGATGATGATGGGCAACG
Rat	Cdkn2a R	TGGTGGGGTCCTCGCAGTTC
Rat	Cdkn1a R	TCCTGGTGATGTCCGACCTGTTC
Rat	Cdkn1a F	GCGGCTCAACTGCTCACTGTC
Rat	Il1b R	CTCACAGCAGCATCTCGACAAGAG
Rat	Il1b F	TCCACGGGCAAGACATAGGTAGC
Rat	Tnf R	ATGGGCTCCCTCTCATCAGTTCC
Rat	Tnf F	GCTCCTCCGCTTGGTGGTTTG
Rat	Il6 R	ACTTCCAGCCAGTTGCCTTCTTG
Rat	Il6 F	TGGTCTGTTGTGGGTGGTATCCTC
Rat	GAPDH F	AGAACATCATCCCTGCATCC
Rat	GAPDH R	CACATTGGGGGTAGGAACAC
Mouse	Cdkn2a F	CCGATTCAGGTGATGATGATGGG
Mouse	Cdkn2a R	CGGGCGGGAGAAGGTAGTG
Mouse	Cdkn1a F	AAAGTGTGCCGTTGTCTCTTCG
Mouse	Cdkn1a R	AAGTCAAAGTTCCACCGTTCTCG
Mouse	Trp53 F	TGAACCGCCGACCTATCCTTAC
Mouse	Trp53 R	GCACAAACACGAACCTCAAAGC
Mouse	Il1b F	TCGCAGCAGCACATCAACAAG

Table S2 (continued)

**Species**	**Primer**	**Sequence (5’ to 3’)**
Mouse	Il1b R	TCCACGGGAAAGACACAGGTAG
Mouse	Tnf F	CACGCTCTTCTGTCTACTGAACTTC
Mouse	Tnf R	CTTGGTGGTTTGTGAGTGTGAGG
Mouse	Il6 F	GAGAGGAGACTTCACAGAGGATATC
Mouse	Il6 R	TCATTTCCACGATTTCCCAGAGAAC
Mouse	GAPDH F	AAATGGTGAAGGTCGGTGTG
Mouse	GAPDH R	AGGTCAATGAAGGGGTCGTT
