# Supplementary material for: Exploring the Therapeutic Effect of Polygonatum cyrtonema Polysaccharides in Reversing D-Galactose (D-Gal)-Mediated Cardiac Aging
Source: Nutrients. 2026 Apr 28;18(9):1390. doi: 10.3390/nu18091390 (PMC13165075; doi:10.3390/nu18091390)
Supplement: Supplementary file 1 [file nutrients-18-01390-s001.zip › Supplementary Figure S1.pdf]

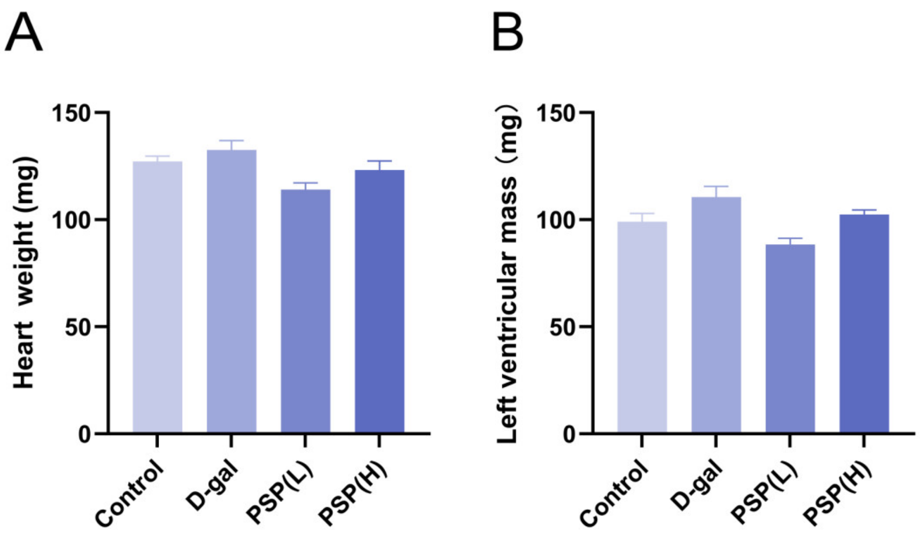

Figure S1. Heart weight and left ventricular mass. (A) Heart weight. (B) Left ventricular mass. The data are shown as the mean ± SEM, n = 5/group.
